# First Evidence of a Protective Effect of Plant Bioactive Compounds against H_2_O_2_-Induced Aconitase Damage in Durum Wheat Mitochondria

**DOI:** 10.3390/antiox9121256

**Published:** 2020-12-10

**Authors:** Maura N. Laus, Mario Soccio

**Affiliations:** Department of Agriculture, Food, Natural Resources and Engineering (DAFNE), University of Foggia, via Napoli, 25, 71122 Foggia, Italy; maura.laus@unifg.it

**Keywords:** aconitase, antioxidant capacity, plant mitochondria, durum wheat, oxidative damage, phytochemicals, ROS

## Abstract

In order to contribute to the understanding of the antioxidant behavior of plant bioactive compounds with respect to specific subcellular targets, in this study, their capability to protect aconitase activity from oxidative-mediated dysfunction was evaluated for the first time in plant mitochondria. Interest was focused on the Krebs cycle enzyme catalyzing the citrate/isocitrate interconversion via *cis*-aconitate, as it possesses a [4Fe-4S]^2+^ cluster at the active site, making it an early and highly sensitive target of reactive oxygen species (ROS)-induced oxidative damage. In particular, the effect on the aconitase reaction of five natural phenols, including ferulic acid, apigenin, quercetin, resveratrol, and curcumin, as well as of the isothiocyanate sulforaphane, was investigated in highly purified mitochondria obtained from durum wheat (DWM). Interestingly, a short-term (10 min) DWM pre-treatment with all investigated compounds, applied at 150 µM (75 µM in the case of resveratrol), completely prevented aconitase damage induced by a 15 min exposure of mitochondria to 500 µM H_2_O_2_. Curcumin and quercetin were also found to completely recover DWM-aconitase activity when phytochemical treatment was performed after H_2_O_2_ damage. In addition, all tested phytochemicals (except ferulic) induced a significant increase of aconitase activity in undamaged mitochondria. On the contrary, a relevant protective and recovery effect of only quercetin treatment was observed in terms of the aconitase activity of a commercial purified mammalian isoform, which was used for comparison. Overall, the results obtained in this study may suggest a possible role of phytochemicals in preserving plant mitochondrial aconitase activity, as well as energy metabolism, against oxidative damage that may occur under environmental stress conditions. Further investigations are needed to elucidate the physiological role and the mechanism responsible for this short-term protective effect.

## 1. Introduction

In the past few decades, a great deal of attention has been focused on natural compounds of vegetal origin, often referred to as phytocompounds or phytochemicals. They include chemically and structurally different plant secondary metabolites, divided into the major classes of polyphenols, terpenes, betalains, organ sulfur compounds, etc. [1]. They are synthesized and accumulated by plants to perform their normal physiological functions, as well as to respond to various environmental stresses and to protect themselves from microbial pathogens, invading insects, herbivorous animals, and competitor plants [1,2]. In addition to the advantages for plant development and survival, it is widely accepted that the regular consumption of foods rich in phytochemicals can also exert a beneficial role in extending and/or improving human health [1]. Many beneficial effects of phytochemicals on both plant growth and human health are attributable to their ability to counteract reactive xygen species (ROS), by both direct (free radical scavenging, singlet oxygen quenching, hydroperoxide inactivation, etc.) and indirect (up-regulation of antioxidant and detoxifying enzymes and modulation of redox signaling and gene expression) antioxidant mechanisms [3].

In order to investigate the antioxidant mechanisms of plant-derived compounds and plant extracts, a considerable number of chemical assays for in vitro measurement of the antioxidant capacity (AC) have been developed [3,4]. Since these methods show the common limit of highlighting only one or a few antioxidant functions [5], an AC evaluation of plant metabolites should not be concluded based on a single test; instead, an adequate combinatory application of different in vitro AC assays is recommended [4,5]. 

However, in vitro AC assessment cannot reflect the in vivo conditions [3,6,7,8]. In light of this, to obtain more advanced information on the antioxidant efficacy of plant metabolites, an evaluation of the effects exerted at a subcellular level is worthwhile. Among subcellular compartments, mitochondria represent an interesting potential target of antioxidant action. These specialized organelles play prominent roles in cell metabolism and energy generation, as well as in the regulation of cell survival/apoptosis and in the maintenance of cellular antioxidant/oxidant homeostasis, also being one of the main intracellular sites of ROS generation [9,10]. With respect to this last point, plant mitochondria are exposed to high ROS production due to the high oxygen concentration in plant cells, but they also possess very efficient defense systems to counteract ROS-induced damage, thus playing a key role in cell adaptation to environmental stress [11]. As for animal systems, oxidative stress-induced impairment of mitochondrial functions is known to play a crucial role in the pathogenesis of aging and a broad variety of human degenerative diseases [12]. It should also be considered that the effect of different phytochemicals on various mitochondrial functions and processes, including mitochondrial biogenesis, membrane potential, and ROS production, signaling to and from mitochondria, has been largely investigated in animals [9,10], whereas very few reports are available for plant systems [13]. 

The aspect of mitochondrial metabolism on which we focused our interest is the aconitase activity. Mitochondrial aconitase (aconitate hydratase; citrate hydrolyase; EC 4.2.1.3) is a dehydratase that operates in the Krebs cycle by catalyzing the reversible stereo-specific isomerization of citrate to isocitrate via the dehydrated intermediate *cis*-aconitate [14,15]. A different aconitase isoform operates in the cytosol, where it is involved in iron homeostasis in animal cells, as well as in the glyoxylate cycle in oil-storing plant seeds and in the operation of the citrate valve in plant leaves [16,17,18]. Aconitase is an iron–sulfur protein containing a [4Fe–4S]^2+^ cluster at the active site. This explains its high sensitivity to oxidative damage by ROS, as well as nitric oxide (NO) and peroxynitrite, which induce reversible inactivation of the enzyme due to Fe-S cluster oxidation according to the reaction [4Fe-4S]^2+^→[3Fe-4S]^0^ + Fe^2+^ [14,18]. In light of this, aconitase activity can be considered as an early sensor of the redox status and a biomarker for oxidative stress [19]. Interestingly, in animals, Krebs cycle alterations due to the NO targeting of mitochondrial aconitase have recently been demonstrated to play a key role in immunometabolism through regulation of the macrophage function [20]. In plants, the ROS/NO-induced inactivation of mitochondrial aconitase may be a sensitive mechanism able to mediate the signaling pathways required to initiate responses to abiotic and biotic stresses [21]. 

On the other hand, it should also be outlined that the protection of aconitase enzymes may have a critical role in protecting the mitochondrial functions from oxidative stress damage [12].

With respect to this last point, it should be underlined that the potential protective effect of several plant bioactive compounds against mitochondrial aconitase oxidative damage has been the subject of numerous literature studies only involving animal systems. Most of these are in vivo studies, in which aconitase activity was assayed in mitochondria isolated from tissues/organs of animals administered with both the oxidant and antioxidant under study [22,23,24,25,26,27]. Other studies were performed on cell cultures, subjected to long-term treatment with the oxidizing compound/condition and the tested phytochemical, followed by aconitase activity measurement in isolated mitochondria [28]. Conversely, knowledge about the short-term protective effect of bioactive compounds on mitochondrial aconitase dysfunction remains very limited. In fact, only one study is available, in which the effect of 5 min incubation with curcumin of mice colon mitochondria was evaluated before treatment with the inflammatory agent 2,4,6-trinitrobenzene sulfonic acid (TNBS) [29]. To the best of our knowledge, to date, the effect of bioactive compounds on the activity of plant mitochondrial aconitase has not been investigated. 

In light of this, the goal of this study was to gain the first insights into the effect of short-term mitochondria treatment with different phytochemicals on aconitase damage. For this purpose, six structurally different plant metabolites were evaluated, including five natural phenols, such as the hydroxycinnamic acid derivative ferulic acid, the flavonoids apigenin and quercetin, the stilbenoid resveratrol, and the diphenylheptanoid curcumin, as well as the isothiocyanate sulforaphane. The antioxidant properties of these phytocompounds, as well as their health-promoting effects and therapeutic potential, in age-related disorders have been largely investigated by numerous in vitro and in vivo investigations, including human studies and animal models [30,31,32,33,34,35]. In light of the lack of information on the subject in plant systems, the direct effect of the above-mentioned phytochemicals on the aconitase reaction was explored for the first time in highly purified mitochondria obtained from durum wheat (*Triticum durum* Desf.). This is a cereal crop largely cultivated in semiarid Mediterranean regions, where it faces many environmental stresses that can trigger several biochemical, physiological, and morphological changes [36,37,38,39], as well as an oxidative stress condition at a cellular/subcellular level [11]. With respect to this last point, durum wheat mitochondria (DWM) have been extensively studied, since they have been demonstrated to be a target of ROS [40], as well as to actively participate in the cell defense against oxidative stress [11,41,42]. Additionally, for this plant species, an isolation procedure proving the high yield of pure and intact mitochondria from etiolated seedlings is also available [43]. For a comparison with DWM-aconitase, the enzymatic activity of a commercially available mammalian isoform (m-aconitase) was also evaluated. Oxidative damage to both aconitase activity sources was imposed by H_2_O_2_ treatment, and the protective and recovery effects of treatment with the phytochemicals under study were evaluated. 

## 2. Materials and Methods 

### 2.1. Chemicals and Plant Materials

All chemicals at the highest commercially available purity were purchased from Sigma-Aldrich Co. (St. Louis, MO, USA). For AC determinations, phytochemicals were dissolved in ethanol, and for aconitase activity assays, stock solutions of phytochemicals were prepared in dimethylsulfoxide (DMSO). Certified seeds of durum wheat (*Triticum durum* Desf., cv Ofanto) were kindly supplied by the CREA-Cereal Research Centre (Foggia, Italy). 

### 2.2. In Vitro Antioxidant Capacity (AC) Determination by Means of Oxygen Radical Absorbance Capacity (ORAC) and Trolox Equivalent Antioxidant Capacity (TEAC) Methods 

#### 2.2.1. ORAC Method 

The ORAC assay measures the chain-breaking capacity against peroxyl radicals, by evaluating the capability of antioxidants to inhibit the quenching reaction of a fluorescent probe induced by peroxyl radicals generated by 2,2’-azobis(2-amidinopropane) (AAPH) thermal decomposition. In particular, the ORAC protocol described in Ou et al. [44], using the 3’,6’-dihydroxyspiro[isobenzofuran-1[3H], 9’[9H]-xanthen]-3-one (fluorescein, FL) as a probe, and modified as reported in Laus et al. [45] and Soccio et al. [46], was applied by using a CLARIOstar-BMG Labtech microplate reader. Every working well of a 96-well plate contained the assay mixture (final volume of 0.2 mL), consisting of 75 mM Na-phosphate buffer (pH 7.4), 10 nM FL, and an appropriate concentration of the tested phytochemical. The reaction was started by adding 40 mM AAPH and FL fluorescence intensity decay was monitored at 37 °C at the excitation and emission wavelengths of 483 nm (bandwidth 14 nm) and 530 nm (bandwidth 30 nm), respectively. Since phytochemicals were reconstituted in ethanol, each well contained a constant volume of 0.5 μL ethanol. To quantify AC, the area under the fluorescence decay kinetic curve was calculated.

#### 2.2.2. TEAC Method

The TEAC assay evaluates the capability of antioxidants to scavenge the 2,2’-azinobis-(3-ethylbenzothiazoline-6-sulphonate) (ABTS^+^) radical cation, by measuring the decrease in ABTS^+^ absorbance at 734 nm (A_734_). In particular, the TEAC protocol described in Re et al. [47] and modified as described in Laus et al. [48] was applied. The ABTS∙^+^ radical cation solution was generated by ABTS oxidation with potassium persulfate and, before use, diluted with 5 mM Na-phosphate buffer (pH 7.4). Since phytochemicals were reconstituted in ethanol, a constant concentration of ethanol (10%) was maintained in the assay mixture. The (%) decrease of A_734_ measured after 5 min incubation of the tested phytochemical with the ABTS^+^ diluted solution was calculated with respect to A_734_ of the uninhibited radical cation solution and used to quantify AC.

For both AC methods, measurements were carried out in triplicate for three different amounts of sample and AC was quantified by means of a dose–response curve obtained with (±)-6-hydroxy-2,5,7,8-tetramethylchromane-2-carboxylic acid (Trolox) as a standard antioxidant.

### 2.3. Durum Wheat Mitochondria (DWM) Isolation

DWM were purified from 72-h-old etiolated seedlings, as reported in Pastore et al. [43]. The grinding and washing buffers were (i) 0.5 M sucrose, 4 mM cysteine, 1 mM EDTA, 30 mM Tris-HCl (pH 7.50), 0.1% (*w*/*v*) defatted bovine serum albumin (BSA), and 0.6% (*w*/*v*) polyvinylpyrrolidone (PVP)-360, and (ii) 0.5 M sucrose, 1 mM EDTA, 10 mM Tris-HCl (pH 7.40), and 0.1% (*w*/*v*) defatted BSA, respectively. Washed mitochondria were subjected to isopycnic centrifugation in a self-generating density gradient containing 0.5 M sucrose, 10 mM Tris-HCl (pH 7.20), and 28% (*v*/*v*) Percoll (colloidal PVP coated silica, Sigma-Aldrich), in combination with a linear gradient of 0% (top) to 10% (bottom) PVP-40 [49], in order to obtain the purified mitochondrial fraction. This protocol allows mitochondria showing a high purity, high intactness of inner and outer membranes, and good functionality to be obtained [43,50,51,52]. The mitochondrial protein content was determined by the method of Lowry, modified according to Harris [53], using BSA as a standard.

### 2.4. Aconitase Activity Assay

The aconitase enzymatic activity was evaluated by a spectrophotometric assay based on the aconitase/isocitrate dehydrogenase coupled reactions, as reported by Baumgart and Bott [54], with minor modifications. In particular, the conversion of citrate into isocitrate catalyzed by aconitase was monitored by following the absorbance increase at 340 nm at 25 °C due to NADPH (ε_340_ = 6.22 mM^−1^ cm^−1^) generation during the oxidative decarboxylation of isocitrate catalyzed by the coupled isocitrate dehydrogenase reaction. A SpectraMax^®^ M5 Multimode Plate Reader (Molecular Devices, Wokingham, UK) and 96-well plates were used for measurements. As for aconitase activity assessment in the DWM fraction, in order to lyse mitochondria and release aconitase enzymes, DWM proteins (150 μg) were preliminarily lysed in the well by 5 min incubation in an assay medium containing 100 mM Tris–HCl (pH 8.0) and 0.1% (*v*/*v*) Triton X-100. Then, the reaction was started by adding a mixture containing trisodium-citrate, NADP^+^, MnCl_2_, and a commercially available isocitrate dehydrogenase (NADP) isoform, in order to obtain, in a final volume of 200 μL per well, final concentrations equal to 20 mM, 1 mM, 5 mM, and 0.06 enzymatic units (E.U.), respectively. For studying the dependence of DWM-aconitase activity on the protein amount, DWM proteins were assayed in the 30–300 μg range; for determination of the kinetic constants, the citrate concentration was varied in the 0.5–20 mM range. The aconitase reaction rate was calculated as the highest slope to the progress curve.

For a comparison with DWM-aconitase, the enzymatic assay was also performed by using 250 μg of a commercially available mammalian isoform (m-aconitase) from a porcine heart. Since mammalian aconitase enzymes need the externally supplied iron and cysteine for restoring activity lost after purification [18], a preliminary activation of the m-aconitase enzyme was performed in each experiment by preparing 2.5 μg/μL enzyme solution in 100 mM Tris–HCl (pH 8.0) containing 2.5 mM cysteine and 25 μM (NH_4_)_2_Fe(SO_4_)_2_ and incubating the mixture for one hour on ice. The activated m-aconitase was used within 30–60 min.

To evaluate the effect of ROS on aconitase activity, different concentrations of H_2_O_2_ (0.25, 0.5, and 0.75 mM) were applied for different incubation times (5, 10 and 15 min) on m-aconitase. As for DWM-aconitase, the effect of 15 min treatment with H_2_O_2_ ranging from 0.25 to 1 mM was tested. The H_2_O_2_-induced damage to aconitase activity was calculated as the (%) decrease of the reaction rate measured after H_2_O_2_ treatment with respect to that of the control reaction, i.e., the reaction measured at time 0 (without the incubation of proteins in the assay mixture) and in the absence of H_2_O_2_.

In the experiments aimed at evaluating the protective effects of phytochemicals against the H_2_O_2_-induced damage, a pre-treatment of m-aconitase for 10 min with the investigated phytochemical in the 50–150 μM range (25–75 μM for resveratrol) was carried out, followed by 15 min treatment with H_2_O_2_ (750 and 250 μM in Figure 3 and Figure 5, respectively), after which the enzymatic activity was measured. As for DWM-aconitase, mitochondria were pre-treated for 10 min with the tested phytocompound at 150 μM (75 μM in the case of resveratrol), and a 15 min treatment with H_2_O_2_ 500 μM was then performed. The protective effect of phytochemicals was calculated as the (%) increase of the reaction rate measured after treatment with both phytochemicals and H_2_O_2_ with respect to the rate obtained after H_2_O_2_ exposure.

The recovery effect of phytochemicals following H_2_O_2_-induced aconitase damage was evaluated by treating m-aconitase and DWM for 15 min with 750 and 500 μM H_2_O_2_, respectively, and by applying phytocompounds at 150 μM (75 μM in the case of resveratrol) in the last 5 min of H_2_O_2_ incubation.

In all experiments involving phytocompounds, since they were dissolved in DMSO, each well contained a constant volume of 5 μL DMSO.

### 2.5. Statistical Analysis

Statistical analysis was performed by using the StatSoft STATISTICA 7 software. The normal distribution of data of Table 1 and Figures 3–6 was verified by using the Shapiro–Wilk test and homogeneity of variances was verified by Bartlett’s test. Data of Table 1 and Figures 5 and 6 were submitted to a “one-factor” analysis of variance (ANOVA) model. Data of Figures 3 and 4 were submitted to a “two-factor” ANOVA analysing the effect of H_2_O_2_ and phytochemical treatments and their interaction on aconitase activity. Following ANOVA, the multiple comparison post hoc Duncan’s test was applied to assess differences between multiple group means at 0.05 (Figures 3–6) and 0.01 (Table 1) *p* levels of significance.

## 3. Results and Discussion

### 3.1. In Vitro Antioxidant Capacity (AC) of Pure Phytochemicals

In order to ascertain the direct antioxidant effect of the five different natural phenols under study, as well as the isothiocyanate sulforaphane, in vitro AC was preliminarily investigated using the well-known TEAC and ORAC methods. These AC assays were chosen as they use different reaction mechanisms: TEAC is mainly based on redox reactions involving single electron transfer (SET) between ABTS^+^ radical and antioxidant species, while in the ORAC assay, the FL oxidation by peroxyl radicals involves hydrogen atom transfer (HAT) reactions. The results of AC measurements are reported in Table 1.

Both TEAC and ORAC protocols highlighted remarkable ABTS∙^+^ and peroxyl radical scavenging activities of the tested phytochemicals, respectively. TEAC measured the highest AC value for quercetin, followed by the other compounds, according to the following rank: resveratrol > apigenin > curcumin > ferulic acid. As expected, based on the different reaction mechanisms, a different behavior was observed for the ORAC method. This assay provided AC values about 1.5-4-fold higher than those of the TEAC method, with the highest and lowest ones being observed for resveratrol and ferulic acid, respectively. Overall, the TEAC and ORAC measurements confirm the high antioxidant properties of the tested phenolic compounds, in agreement with previous literature data [44,47,55,56]. As for sulforaphane, both AC assays were not able to evaluate any in vitro AC for this compound within the investigated range. This is in agreement with the incapability of this isothiocyanate compound to exert a direct antioxidant action, despite a wide range of other beneficial effects having been reported in animal systems, including indirect antioxidant effects by the induction of antioxidants and phase II detoxifying enzymes [23,57], as well as anti-inflammatory and anti-carcinogenic effects [57].

### 3.2. Induction of H_2_O_2_ Damage to Aconitase Activity

As stated in the introduction, the aim of this paper is to evaluate the effect of short-term treatment with different phytochemicals on H_2_O_2_-induced damage on DWM-aconitase activity. For this purpose, the enzymatic activity was evaluated by an aconitase/isocitrate dehydrogenase coupled reaction-based assay, during which a preliminary kinetic characterization of the aconitase reaction was performed (data not shown). In particular, the DWM-aconitase activity exhibited a behavior that was linearly dependent on the DWM amount in the range from 37.5 to 300 μg and a hyperbolic relationship for the citrate concentration according to the Michaelis-Menten equation, with Km for citrate and Vmax values equal to 4.8 ± 0.9 mM and 29 ± 1 nmol min^−1^ mg^−1^ of DWM protein, respectively.

**Figure 1:**
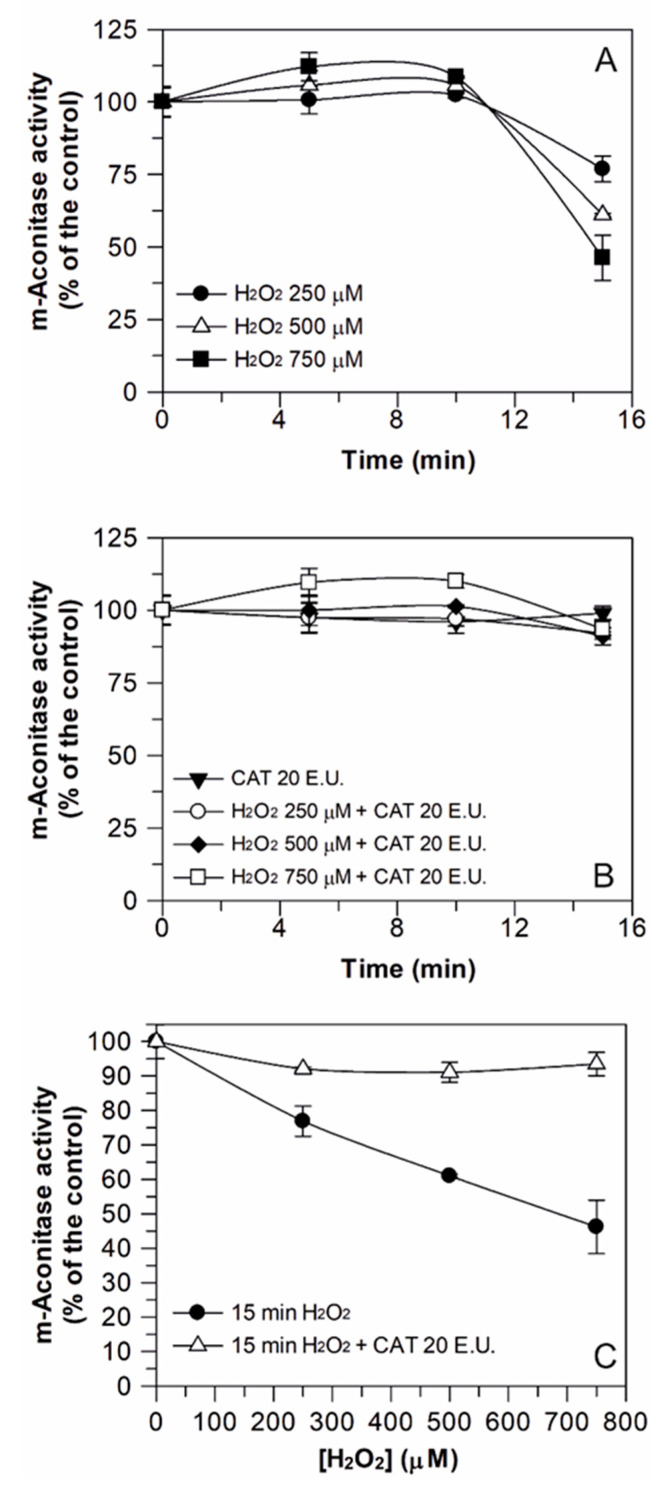
Effect of H_2_O_2_ treatment on m-aconitase activity, evaluated in both the presence and absence of catalase. Measurements were carried out as described in Methods by incubating m-aconitase (250 μg) for different times (5, 10, and 15 min) in 100 mM Tris-HCl pH 8.0 buffer containing different H_2_O_2_ concentrations (250, 500, and 750 μM), both in the absence and presence of 20 E.U. catalase (CAT). Activity values are reported as a percentage of the control (i.e., the reaction measured at time 0 in the absence of both H_2_O_2_ and CAT) and represent the mean of three independent experiments ± SD. In (**A**) and (**B**), the dependence of m-aconitase activity on the incubation time is reported for each H_2_O_2_ concentration tested in both the absence and presence of CAT, respectively. In (**C**), the effect of 15 min treatment of m-aconitase with different H_2_O_2_ concentrations is shown, as evaluated in both the absence and presence of CAT. In this set of experiments, m-aconitase activity under control conditions was equal to 19 ± 1 (SD, *n* = 3) nmol·min^−1^·mg^−1^·of protein.

To define the experimental conditions able to induce damage to aconitase activity, preliminarily, the effect of different H_2_O_2_ concentrations (250, 500, and 750 μM), as well as different incubation times (5, 10, and 15 min), on m-aconitase activity was evaluated (Figure 1). Firstly, m-aconitase activity was shown to remain rather constant over 15 min incubation under control conditions (data not shown). As presented in Figure 1A, H_2_O_2_ did not affect m-aconitase activity up to 10 min incubation, regardless of the H_2_O_2_ concentration used. On the contrary, after 15 min incubation, a decrease of enzyme activity was observed, whose magnitude was proportional to the H_2_O_2_ concentration. When 20 E.U. of the H_2_O_2_ scavenging enzyme catalase (CAT) was preliminarily added to the same reaction mixture, enzyme inactivation was totally prevented (Figure 1B), confirming that damage really depends on H_2_O_2_. CAT was verified to have no effect on the enzyme activity. Data from Figure 1A,B were reorganized as reported in Figure 1C, in which only the data referring to the enzyme activity measured after 15 min incubation with H_2_O_2_ are reported. A progressive decrease of enzyme activity up to about 55% in the presence of 750 μM H_2_O_2_ was clearly observed, which was totally prevented by CAT.

**Figure 2:**
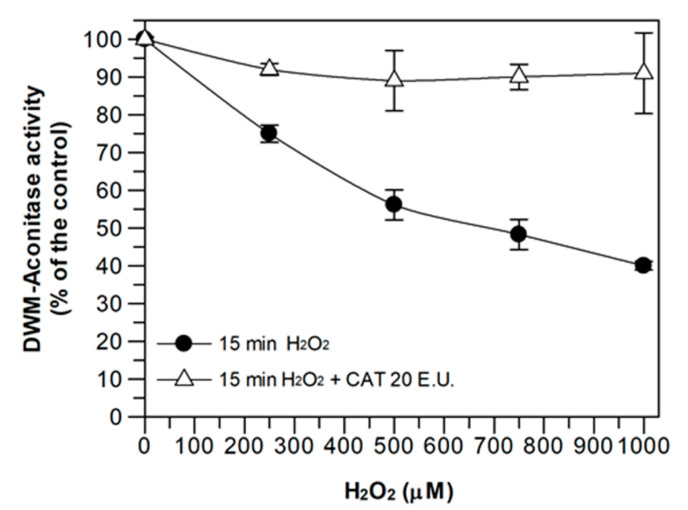
Effect of H_2_O_2_ treatment on durum wheat mitochondria (DWM)-aconitase activity, evaluated in both the presence and absence of catalase. Measurements were carried out as described in Methods, by incubating DWM proteins (150 μg) for 15 min in a reaction medium containing 100 mM Tris–HCl pH 8.0, 0.1% (*v*/*v*) Triton X-100, and different H_2_O_2_ concentrations (250, 500, 750, and 1000 μM), both in the absence and presence of 20 E.U. catalase (CAT). Aconitase activity values are expressed as a percentage of the control (i.e., the reaction measured at time 0 in the absence of both H_2_O_2_ and CAT) and reported as the mean value ± SD (*n* = 3). The dependence of DWM-aconitase activity on the H_2_O_2_ concentration is reported, as evaluated in both the absence and presence of CAT. In this set of experiments, DWM-aconitase activity under control conditions was equal to 28 ± 2 (SD, *n* = 3) nmol·min^−1^·mg^−1^of protein.

A similar behavior was obtained when DWM-aconitase was assayed (Figure 2). In this case, only the effect of 15 min of H_2_O_2_ exposure was evaluated. Incubation with different H_2_O_2_ concentrations (250–1000 μM) induced a progressive inhibition of the enzyme activity up to about 60% in the presence of 1 mM H_2_O_2_. Similar to m-aconitase, the H_2_O_2_ damage was totally prevented by CAT, thus confirming that, also in this case, the inactivation really depends on H_2_O_2_. Once again, CAT was verified to have no effect on the mitochondrial enzyme activity and the DWM-aconitase activity under control conditions remained constant for 15 min.

The results of Figure 1 and Figure 2 indicate that the inhibition of aconitase activity by H_2_O_2_ was a function of both the incubation time and H_2_O_2_ concentration and confirm, once again, that mitochondrial aconitase represents a protein responsive to ROS-mediated oxidative damage [18]. In fact, at external H_2_O_2_ concentrations equal to 750 and 500 μM, 15 min incubation halved the enzyme activity of both m-aconitase and DWM-aconitase, respectively. On the other hand, the data of Figure 2 may suggest a lower sensitivity of DWM-aconitase with respect to other plant species. In fact, the activity of aconitase isolated from potato tuber mitochondria was found to be completely blocked by 300 and 500 μM H_2_O_2_ in about 6 and 3 min, respectively [14]. Moreover, the activity of mitochondrial aconitase isoform purified from 4-day-old maize scutella showed complete inactivation at 300 μM H_2_O_2_ [18]. This different H_2_O_2_ sensitivity could be explained considering that potato tuber and maize scutella aconitase isoforms were highly purified mitochondrial enzymes, while DWM-aconitase activity was assayed within its biological environment (i.e., the mitochondrial suspension), where the occurrence of other reactions involving H_2_O_2_ cannot be excluded.

Based on the results of Figure 1 and Figure 2, in the subsequent experiments on DWM-aconitase and m-aconitase, a 15 min incubation with 500 and 750 μM of H_2_O_2_ was respectively performed, as these treatments produced comparably significant damage, corresponding to 50 ± 5 (%) enzyme inactivation, in both aconitase sources, without completely compromising the enzymatic activity.

### 3.3. Effect of Phytochemicals on the H_2_O_2_-Induced Damage of Aconitase

**Figure 3:**
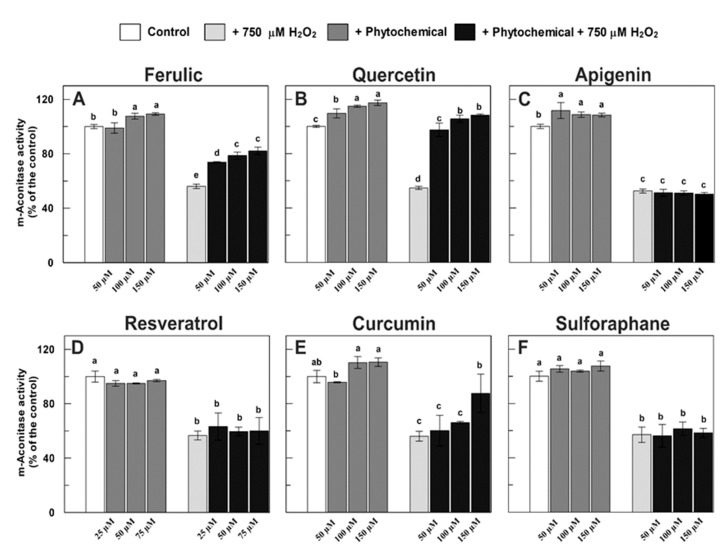
Effect of different phytochemicals on m-aconitase activity, both untreated and treated with 750 μM H_2_O_2_. Measurements were carried out as described in Methods by incubating m-aconitase (250 μg) for 10 min with different phytochemicals at three different concentrations and/or for 15 min with 750 μM H_2_O_2_. Activity values are expressed as a percentage of the control (i.e., the reaction measured at time 0 in the absence of both H_2_O_2_ and phytochemicals) and reported as the mean value ± SD (*n* = 3). Within each graph, means with no lowercase letter in common are statistically different at a 0.05 *p* level of significance, according to the post hoc Duncan’s test, following ANOVA. Means with at least one common lowercase letter are not significantly different at *p* ≤ 0.05. In this set of experiments, m-aconitase activity under control conditions ranged from 17.1 to 18.9 nmol·min^−1^·mg^−1^ of protein.

In Figure 3, the possible protective effect of the investigated phenolic compounds, as well as the isothiocyanate sulforaphane, against the H_2_O_2_-induced damage of m-aconitase activity was reported. The enzyme was pre-incubated for 10 min with the different investigated phytochemicals, followed by 15 min treatment with 750 μM H_2_O_2_ (H_2_O_2_-treated m-aconitase) or not (H_2_O_2_-untreated m-aconitase). Based on literature indications [58], in the present study, all tested compounds were evaluated in the 50–150 μM range, except for resveratrol, which was tested in the 25–75 μM range due to its higher absorbance at 340 nm. All tested phytocompounds were proven to have no effect on the coupled enzymatic assay.

In accordance with the results reported in Figure 1, the incubation of m-aconitase with H_2_O_2_ almost halved the enzyme activity (Figure 3). Interestingly, when m-aconitase was pre-incubated with ferulic acid (Figure 3A), partial protection of the enzyme activity was obtained, reaching up to about 80% of the control (corresponding to a protective effect of about 45% of the oxidative damage) in the presence of 100–150 μM ferulic acid. Moreover, statistically significant but very low (up to about 10%) direct activation was observed in H_2_O_2_-untreated m-aconitase at 100–150 μM ferulic acid.

Contrarily, quercetin was able to exert a more evident positive effect (Figure 3B). The H_2_O_2_ damage of m-aconitase was completely prevented by 50 μM quercetin and its positive effect was even more evident at higher quercetin concentrations. Interestingly, a little direct enzyme activation, up to about 20% in the presence of 150 μM quercetin, was also observed in untreated m-aconitase. As for apigenin, no protective effect on H_2_O_2_-damaged m-aconitase was obtained, despite a very low direct activation of the enzyme (about 10%) being measured (Figure 3C). In contrast, both resveratrol and sulforaphane were unable to exert an effect on both H_2_O_2_-damaged and untreated m-aconitase (Figure 3D,F), whereas curcumin exerted only a small protective role, up to about 40% of the damage at 150 μM (Figure 3E).

**Figure 4:**
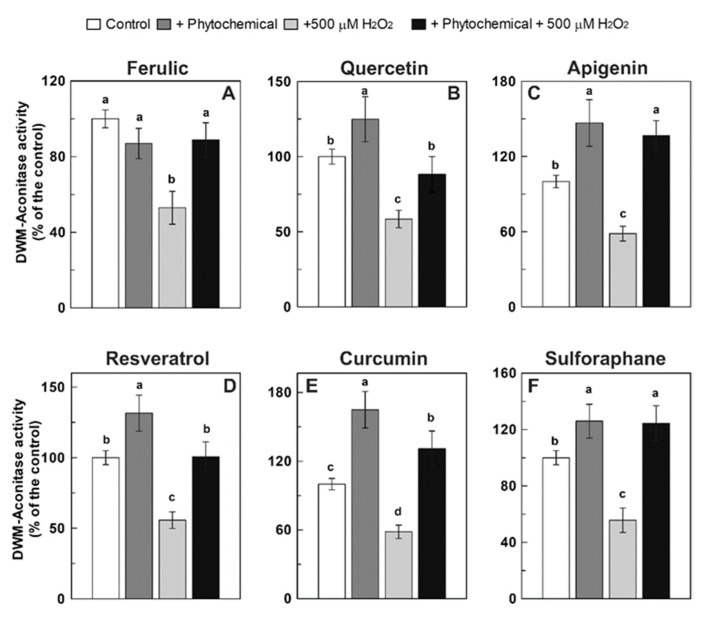
Effect of different phytochemicals on DWM-aconitase activity, both untreated and treated with 500 μM H_2_O_2_. Measurements were carried out as described in Methods by incubating DWM (150 μg) for 10 min with different phytochemicals and/or for 15 min with 500 μM H_2_O_2_. All compounds were tested at a concentration of 150 μM, except for resveratrol, which was applied at 75 μM. Activity values are expressed as a percentage of the control (i.e., the reaction measured at time 0 in the absence of both H_2_O_2_ and phytochemicals) and reported as the mean value ± SD (*n* = 3). Within each graph, means with no lowercase letter in common are statistically different at a 0.05 *p* level of significance, according to the post hoc Duncan’s test, following ANOVA. Means with at least one common lowercase letter are not significantly different at *p* ≤ 0.05. In this set of experiments, DWM-aconitase activity under control conditions ranged from 27.6 to 30.5 nmol·min^−1^·mg^−1^ of protein.

A very different behavior was obtained when the tested phytochemicals were evaluated on the DWM-aconitase (Figure 4). In this case, all tested compounds were studied at 150 μM (75 μM in the case of resveratrol). In line with the results of Figure 2, H_2_O_2_ treatment induced damage of the enzyme activity of about 50–55%. The pre-incubation of DWM with 150 μM ferulic acid almost completely prevented aconitase activity in H_2_O_2_-treated mitochondria, thus indicating the capability of this phenolic acid to prevent H_2_O_2_-dependent inhibition of the enzyme (Figure 4A). Quercetin pre-treatment ameliorated—up to about 90% of the control—the H_2_O_2_-induced suppression of aconitase activity (Figure 4B), while activity in DWM exposed to both H_2_O_2_ and apigenin was even 35% higher than that measured in untreated DWM (Figure 4C). Interestingly, when DWM were treated with 150 μM of both quercetin and apigenin alone, a remarkable activation—up to about 25% and 50%—of aconitase activity was found, respectively (Figure 4B,C). Resveratrol alone, applied at a concentration equal to 75 μM, was able to completely protect the enzyme against the H_2_O_2_-dependent damage and to increase aconitase activity by about 30% (Figure 4D). Interestingly, 150 μM curcumin provided the highest activation of DWM-aconitase, being 65% higher than the control (Figure 4E); as observed for apigenin, the remarkable aconitase activation by curcumin was also maintained after DWM treatment with H_2_O_2_. Finally, sulforaphane was able to totally prevent the H_2_O_2_-induced DWM-aconitase damage and activate the enzyme up to about 25% in both the absence and presence of H_2_O_2_.

**Figure 5:**
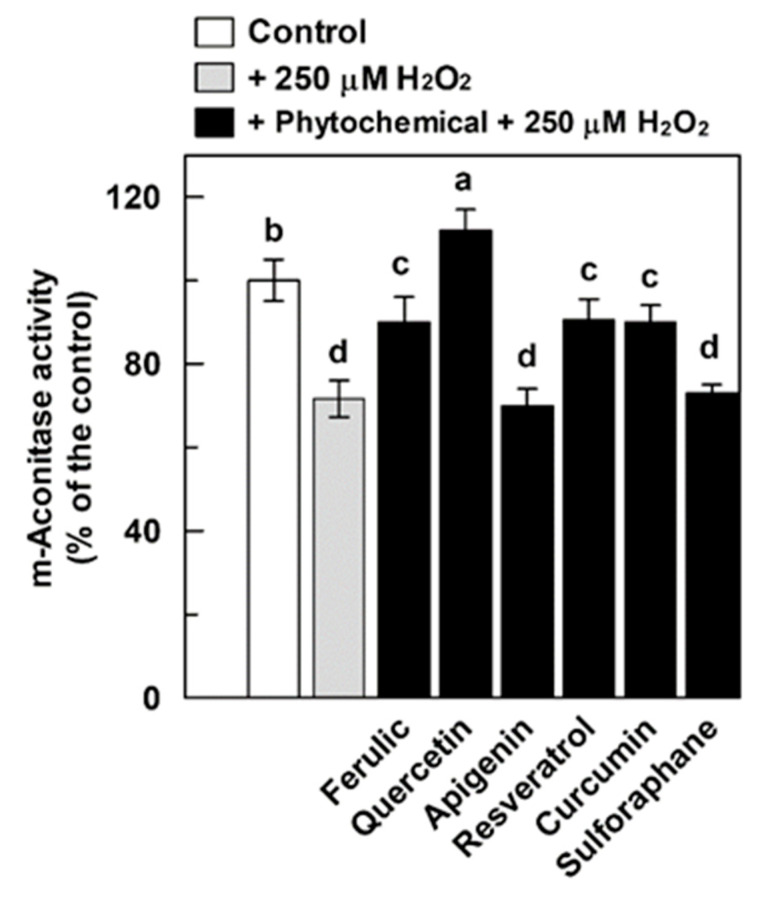
Protective effect of different phytochemicals on m-aconitase activity treated with 250 μM H_2_O_2_**.** Measurements were carried out as described in Methods by incubating m-aconitase (250 μg) for 10 min with different phytochemicals applied at 150 μM (75 μM in the case of resveratrol) and for 15 min with H_2_O_2_. Activity values are expressed as a percentage of the control (i.e., the reaction measured at time 0 in the absence of both H_2_O_2_ and phytochemicals) and reported as the mean value ± SD (*n* = 3). Means with no lowercase letter a–d in common are statistically different at a 0.05 *p* level of significance, according to the post hoc Duncan’s test, following ANOVA. Means with at least one common lowercase letter are not significantly different at *p* ≤ 0.05. In this set of experiments, m-aconitase activity under control conditions was equal to 18 ± 1 (SD, *n* = 3) nmol·min^−1^·mg^−1^ of protein.

The protective effect of phytochemicals was also evaluated against minor damage of aconitase activity (Figure 5). For this purpose, we only focused on m-aconitase, since the complete protection of DWM-aconitase by all tested phytochemicals had already been observed against 500 μM H_2_O_2_-induced damage. In particular, after a pre-treatment with phytocompounds at 150 μM (75 μM in the case of resveratrol), a 15 min treatment of m-aconitase with 250 μM H_2_O_2_ was performed, as it was able to induce oxidative damage of about 25–30% (also see Figure 1). In accordance with the results of Figure 3, a partial protective effect by ferulic acid and curcumin and more than complete protection by quercetin of the enzyme activity were observed on 250 μM H_2_O_2_-treated m-aconitase. Interestingly, unlike the results reported in Figure 3, a statistically significant protection (about 90% of the control corresponding to about 30% of damage) of the enzyme activity by resveratrol was also observed. Once again, apigenin and sulforaphane were unable to exert any effect. These data suggest that (i) the extent of H_2_O_2_ damage may affect the behavior of the various phytochemicals in a different manner and (ii) the efficacy of the tested compounds may be different, depending on the stoichiometry (H_2_O_2_: phytochemical) adopted.

**Figure 6:**
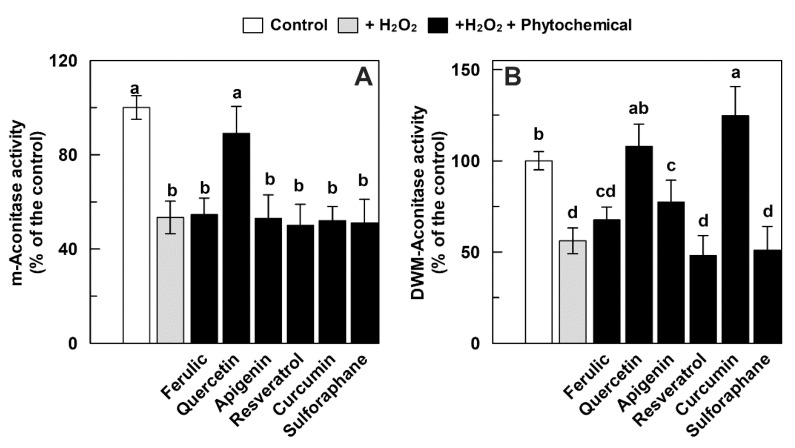
Recovery effect of different phytochemicals on m-aconitase (**A**) and DWM-aconitase (**B**) activities treated with H_2_O_2_. Measurements were carried out as described in Methods by treating m-aconitase (250 μg) for 15 min with 750 μM H_2_O_2_ (**A**) and DWM (150 μg) with 500 μM H_2_O_2_ (**B**) and in the last 5 min of incubation, the different phytochemicals were added at 150 μM (75 μM in the case of resveratrol). Activity values are expressed as a percentage of the control (i.e., the reaction measured at time 0 in the absence of both H_2_O_2_ and phytochemicals) and reported as the mean value ± SD (*n* = 3). Means with no lowercase letter in common are statistically different at a 0.05 *p* level of significance, according to the post hoc Duncan’s test, following ANOVA. Means with at least one common lowercase letter a–d are not significantly different at *p* ≤ 0.05. In this set of experiments, m-aconitase and DWM-aconitase activities under control conditions were equal to 18 ± 1 and 30 ± 2 (SD, *n* = 3) nmol·min^−1^·mg^−1^ of protein, respectively.

The tested phytocompounds were also evaluated in relation to their possible role in restoring aconitase activity after the H_2_O_2_ treatment, i.e., when the oxidative damage is already established (Figure 6). Both m-aconitase and DWM were treated with H_2_O_2_ (750 and 500 μM, respectively) for 15 min; the last 5 min of which occurred in the presence of the tested phytochemicals at 150 μM (75 μM in the case of resveratrol). None of the investigated compounds were able to exert a significant positive effect on m-aconitase activity, with the only exception of quercetin, which induced an almost complete recovery of the enzyme activity (Figure 6A). As for DWM-aconitase, ferulic acid, resveratrol, and sulforaphane were unable to exert any positive effect under the set experimental conditions (Figure 6B). On the other hand, apigenin induced only a partial restoration of enzyme activity of about 80% of the control, whereas a more than complete recovery of the H_2_O_2_-damaged DWM-aconitase was observed for quercetin. Interestingly, curcumin was able to not only completely recover the damaged DWM-aconitase activity, but also to activate the enzyme by about 25% (Figure 6B).

On the whole, the information obtained in this study is very interesting and can be considered a novelty with respect to several different aspects, although further investigations are needed to clarify them.

Firstly, in terms of the comparison of the performance of DWM- and m-aconitase enzymes investigated in this study, a very different sensitivity towards the protecting/recovering action of the phytochemicals against H_2_O_2_-induced damage was observed. In fact, the H_2_O_2_-induced damage to DWM-aconitase was prevented by all tested compounds, albeit with a different effectiveness, as well as partially recovered by apigenin and totally by quercetin and curcumin. Moreover, with the only exception of ferulic acid, all tested compounds also exhibited the ability to increase aconitase activity in H_2_O_2_-untreated DWM. On the other hand, only quercetin seems to have significantly important protective and recovery effects on m-aconitase activity, as well as to induce an about 20% activation of undamaged enzyme. Lacking literature information about these aspects, this different behavior could be speculated to partly depend on differences between DWM- and m-aconitase activity assays, which were performed on purified mitochondria preparations in the first case and on highly purified commercial enzyme from a porcine heart in the second one. The use of this commercial purified aconitase enzyme implies the need to externally supply cysteine and iron to restore activity lost during the purification procedure due to the higher sensitivity of the mammalian Fe-S cluster to degradation compared to plant isoforms [18]. It is possible to speculate that the different reaction environment for aconitase activity assessment due to the presence of iron and reducing agents can mask/affect the phytochemical effect in the case of m-aconitase. However, a higher sensitivity to the phytochemical protecting/recovering action of plant aconitases, compared to the animal ones, cannot be excluded, since no literature information is available about this aspect in plant systems, as discussed below.

It should be considered that the results obtained in this study about the short-term protective role of phytochemicals over aconitase activity changes are in agreement with that reported by Mouzaoui et al. [28], showing the capability of 5 min curcumin treatment to restore aconitase activity in TNBS-treated mice colon mitochondria. Moreover, the results of the present study are also in accordance with some literature reports relative to long-term phytochemical treatment performed in animal systems. In particular, these data regard the capability of (i) curcumin to prevent aconitase damage in liver [24,26], heart [25], and kidney [27] mitochondria isolated by treated rats; (ii) sulforaphane to abrogate the H_2_O_2_ effect on mitochondrial aconitase activity in SH-SY5Y cells [28] and to prevent aconitase inactivation in kidney mitochondria isolated from cisplatin-treated rats [22]; and (iii) quercetin to partly ameliorate the suppression of aconitase activity in mice subjected to intense exercise [59].

Compared to these literature data, the present study shows several novelty items, the most relevant of which is that for the first time the phytochemical prevention of oxidative stress-mediated dysfunction of mitochondrial aconitase has been studied in plant systems. To the best of our knowledge, this is also the first study providing evidence about the effect of ferulic, apigenin, and resveratrol on aconitase activity, for which no literature data are available, even in animal systems. In addition, with the only exception of curcumin [29], for the first time, the protecting activity of phytochemicals, including phenolic acids, flavonoids, stilbenes, and isothiocyanate compounds, has been investigated in the present study by the direct and short treatment of H_2_O_2_-damaged DWM-aconitase.

As for the mechanism responsible for the aconitase-damage protecting activity of short-term phytochemical treatment observed in this study, further studies are required to elucidate it. A direct H_2_O_2_ scavenging activity of the investigated compounds is undoubtedly involved. With respect to this point, the capability to efficiently scavenge H_2_O_2_ is widely known for the tested phenolic compounds, including ferulic acid, quercetin, resveratrol, and curcumin [9,47,48,49,50,51,52,53,54,55,56,57,58,59,60,61]. Moreover, in accordance with their high reducing activity against the ABTS∙^+^ radical cation, the capability of the investigated phytochemicals to act as reducing agents, which are reported to be capable of maintaining the reducing groups of the protein and the iron in the reduced state [62], cannot be excluded under the applied experimental conditions. However, the high protecting efficacy observed after DWM pre-treatment with sulforaphane—unable to exert a direct antioxidant action [23]—strongly suggests that the observed effect cannot depend solely on the direct H_2_O_2_ scavenging activity and reducing capacity of the investigated compounds. This is also suggested by the high recovery of aconitase activity shown by quercetin and curcumin, and to a lesser extent, by apigenin on H_2_O_2_-damaged DWM, as well as by quercetin in the case of m-aconitase. In addition, a direct interaction of phytochemicals with the aconitase enzyme could be another mechanism contributing to aconitase protection by short-term phytochemical treatment. The occurrence of this interaction can be suggested by the significant increase of aconitase activity observed after the incubation of H_2_O_2_-undamaged DWM with all tested phytochemicals (except ferulic acid), as well as with quercetin in the case of m-aconitase.

Even with respect to the effect of phytochemicals on untreated aconitase activity, the results provided by the present paper represent a novelty. Only one study is available, reporting a significant increase (30%) of aconitase activity in mitochondria isolated from PC12 cells, which was observed after long-term (24 h) cell treatment with curcumin [12]. To the best of our knowledge, to date, the direct modulation of catalytic activity of mitochondrial aconitases by short-term phytochemical treatment has never been reported in either animals or plants. In light of the absolute lack of information on this issue, the possible chemical mechanisms responsible for direct phytochemical–aconitase interaction remain to be fully elucidated. For this purpose, the possible involvement of the Fe-S cluster or other functional groups of the active site of the aconitase enzyme could be very interesting subjects of future investigations. However, the capability of some plant compounds to act as aconitase enzyme activators could have a relevant physiological significance. A potential role of phytochemicals in preserving aconitase functioning can be suggested, as well as in protecting mitochondrial energy metabolism and in maintaining redox homeostasis, in particular under oxidative stress conditions [12]. This regulation may be particularly interesting for plant systems, in which the accumulation of secondary metabolites, particularly that of phenolic compounds, occurs in vegetative tissues for adaptation to environmental stresses. In light of this, a possible role of plant mitochondrial aconitase as a sensitive mechanism operating in the regulation of plant metabolic networks connecting the Krebs cycle, ROS, and phenolic metabolism under stress conditions can be hypothesized based on the results obtained in this study.

Finally, the results reported in this study also suggest the possibility of using the H_2_O_2_-damage protecting activity of mitochondrial aconitase as a useful tool for AC evaluations of plant bioactive compounds. To do this, the study should be enlarged to different classes of phytocompounds and plant extracts, possibly investigated in a wide concentration range, as well as at different incubation times. This innovative approach could integrate in vitro and in vivo AC studies, thus providing as much information as possible reflecting the in vivo antioxidant response.

## 4. Conclusions

The results of this study confirm that mitochondrial aconitase represents a protein responsive to ROS-induced oxidative damage in durum wheat. Interestingly, for the first time, the capability of several plant secondary metabolites to counteract ROS-dependent aconitase inactivation has been demonstrated in plant mitochondria, and a possible phytochemical–aconitase interaction has been suggested. Hence, this study promotes further research aimed at clarifying the mechanism and physiological role of this protective effect, as well as verifying if it is a property common to other plant mitochondria.

## Figures and Tables

**Table 1:** Antioxidant capacity (AC) of different phytochemicals, as evaluated by Trolox Equivalent Antioxidant Capacity (TEAC) and Oxygen Radical Absorbance Capacity (ORAC) methods.

Phytochemical	AC (μmol Trolox eq./μmol)	
	TEAC	ORAC
**Ferulic acid**	1.52 ± 0.02 ^E^	5.18 ± 0.74 ^c^
**Apigenin**	2.74 ± 0.12 ^C^	8.46 ± 0.73 ^b^
**Quercetin**	4.99 ± 0.30 ^A^	8.71 ± 0.50 ^b^
**Curcumin**	2.30 ± 0.08 ^D^	8.79 ± 0.73 ^b^
**Resveratrol**	4.38 ± 0.14 ^B^	10.45 ± 1.16 ^a^
**Sulforaphane**	N.D.	N.D.

Measurements were performed as described in Methods. In each column, means not sharing any lowercase or uppercase letter are statistically different at 0.05 and 0.01 *p* levels of significance, respectively, according to the post hoc Duncan’s test, following ANOVA. Means with at least one common lowercase a–c or uppercase A–E letter are not significantly different at *p* ≤ 0.05 and *p* ≤ 0.01, respectively. All data are expressed as the mean value ± SD (*n* = 3 independent experiments). N.D. not detected in 50–200 and 10–40 μM ranges for TEAC and ORAC assays, respectively.
